# Prepapillary Vascular Loop Associated with Persistent Hyperplastic Primary Vitreous

**DOI:** 10.1155/2013/259797

**Published:** 2013-05-23

**Authors:** Shinji Makino, Yuko Ohkubo, Hironobu Tampo

**Affiliations:** Department of Ophthalmology, Jichi Medical University, Shimotsuke, 3311-1 Yakushiji, Tochigi 329-0498, Japan

## Abstract

A 66-year-old woman was referred for evaluation of retinal vessel abnormality. She had no visual symptoms. The anterior segment showed a retrolental opacity in her right eye. Fundus examination showed a bilateral prepapillary vascular loop associated with PHPV in her right eye. Prepapillary vascular loops were present in both eyes, although they were much more prominent in the right eye. To our knowledge, there are no reports of prepapillary vascular loops associated with PHPV.

## 1. Introduction

Prepapillary vascular loop formations are uncommon congenital vascular malformations and the majority is of arterial origin [1]. They are usually detected incidentally on routine fundus examination but have been associated with retinal vascular occlusive disease and recurrent vitreous bleeding [2]. Prepapillary vascular loops are quite rare, with the incidence ranging from approximately one in 2000 to one in 9000 patients. Bilaterality occurs in 9–17% of these cases [3].

Persistent hyperplastic primary vitreous (PHPV) is a rare congenital developmental malformation of the eye and is caused by the failure of regression of the primary vitreous characterized by microphthalmia, a shallow anterior chamber, elongated ciliary processes, a posterior subcapsular cataract, and a fibrovascular stalk that extends from the optic disc to the lens to varying degrees [4]. PHPV can occur with other ocular and/or systemic disorders. To our knowledge, there are no reports of prepapillary vascular loops associated with PHPV. Herein, we describe the case of such a patient. 

## 2. Case Report

**Figure 1:**
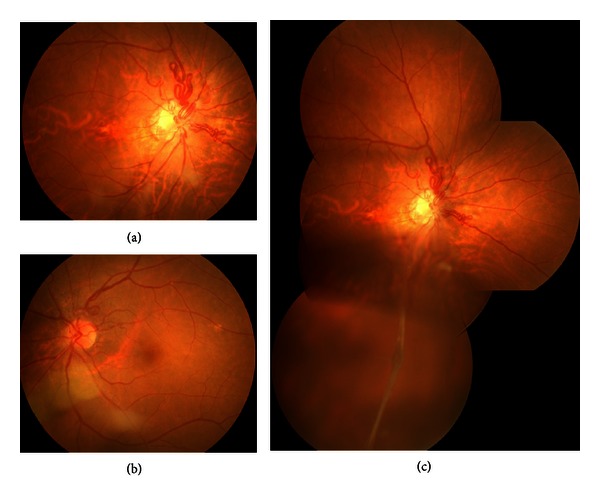
Fundus photographs showing a bilateral prepapillary vascular loop (a, b) and persistent hyperplastic primary vitreous in the right eye (c).

A 66-year-old woman was referred for evaluation of retinal vessel abnormality. She had no visual symptoms. She had a history of hypertension, hyperlipidemia, and cerebral un-ruptured aneurysm. Her visual acuity was 1.0 bilaterally. The anterior segment showed a retrolental opacity in her right eye. Fundus examination showed a bilateral prepapillary vascular loop associated with PHPV in her right eye. Prepapillary vascular loops were present in both eyes, although they were much more prominent in the right eye (Figures 1(a), 1(b), and 1(c)).

## 3. Discussion

Embryologically, the developing part of the front section of the eye receives its nutrients from a hyaloid artery, which is an intraocular vascular system between the retina and crystalline lens [4]. 

During later stages of development, this hyaloid system completely regresses, and its role is gradually replaced by the developing retinal vasculature. However, in some cases, the primary vitreous fails to regress between the third and ninth months of gestation. This results in PHPV.

PHPV can occur with other disorders including Axenfeld-Rieger syndrome, neurofibromatosis, Aicardi syndrome, acute angle closure glaucoma, myopia, neurological abnormalities, retinal tumor, osteoporosis-pseudoglioma syndrome, morning glory syndrome, congenital grouped pigmentation, megalocornea, bilateral retinal vascular hypoplasia, retinopathy of prematurity, fetal adenoma of the pigmented ciliary epithelium, retinoschisis, and orbital lymphangioma [4]. To our knowledge, there are no reports of prepapillary vascular loops associated with PHPV.

Shakin et al. [5] described an anatomic study of prepapillary vascular loop. The loop communicated with the retinal arterial system and did not have an internal elastic lamina. Additionally, the connective tissue matrix of the vascular loop contained less hyaluronic acid than the vitreous. Their findings seem to support the embryologic deviation of prepapillary vascular loops from the retinal arterial system, rather than from the hyaloid artery because of the anatomic separation from the primary vitreous. However, it is not clear whether this case represents the possibility of a relation between the vascular loop and PHPV or an incidental association. Further studies with additional cases are necessary to answer this question.
